# Pediatric COVID-19 in Lesotho and Post-pandemic Implications on Lower Respiratory Infections in Children

**DOI:** 10.7759/cureus.65938

**Published:** 2024-08-01

**Authors:** Kristen S Joseph, Tiiso D Lekhela, Michael R Rose, Lawrence Gersz, More Mungati, Matsosane Shoba, Sello Montsi, Sebaki F Leluma, Lawrence Oyewusi, Bhakti Hansoti, Justine Mirembe, Nicole A Shilkofski, Nyikadzino Mahachi, Eric D McCollum

**Affiliations:** 1 Global Program for Pediatric Respiratory Sciences, Eudowood Division of Pediatric Respiratory Sciences, Pediatrics, Johns Hopkins University School of Medicine, Baltimore, USA; 2 Anesthesia and Critical Care Medicine, Johns Hopkins University School of Medicine, Baltimore, USA; 3 General Practice, Jhpiego, Maseru, LSO; 4 Emergency Medicine, Johns Hopkins University School of Medicine, Baltimore, USA; 5 Research, United States Agency for International Development, Pretoria, LSO; 6 Pediatrics, Johns Hopkins University School of Medicine, Baltimore, USA; 7 International Health, Bloomberg School of Public Health, Johns Hopkins University, Balitmore, USA

**Keywords:** hypoxemia, respiratory tract infections, pediatrics, developing countries, lesotho, covid-19

## Abstract

Background

The United States Agency for International Development (USAID) Reaching Impact, Saturation, and Epidemic Control (RISE) program funded Jhpiego to support the Government of Lesotho’s COVID-19 response, including two national COVID-19 treatment centers. To evaluate the status of post-pandemic pediatric respiratory care in Lesotho, we analyzed pediatric treatment center data and healthcare worker (HCW) performance on pediatric COVID-19 training offered to HCWs at COVID-19 treatment centers.

Methods

We conducted a retrospective cohort study of patients 15 years of age or less hospitalized at two COVID-19 treatment centers in Lesotho from May 1, 2020, to April 30, 2022. Patient data were extracted from hospital files. We used the independent sample t-test, Mann-Whitney U test, or Fisher's exact test to evaluate associations between exposure variables and death. We also assessed differences between pre- and post-training examination scores of three one-day HCW training on pediatric COVID-19 using paired t-tests.

Results

Overall, <15-year-olds comprised 18/1,448 (1.2%) hospitalizations. Twenty-two percent (4/18) of children were hypoxemic (oxyhemoglobin saturation <94%) within the first 24 hours and 44% (8/18) at any point in the hospitalization. Oxygen utilization increased over the two-year period (p=0.004) and all eight children with hypoxemia received oxygen (p<0.001). Four of 18 (22%) patients died. For HCW training, pre- and post-training examinations were completed by 76/82 (92.7%) participants. The overall mean pretraining score was 44.6% (standard deviation (SD) 15.7%). Mean scores improved by an average of 32.2% (95% confidence interval (CI) 27.7%, 36.6%, p<0.001) on the same day post-training examination.

Conclusions

National COVID-19 treatment center data indicate a low burden of severe pediatric COVID-19 disease in Lesotho. However, recognized HCW knowledge gaps suggest deficiencies in identifying and referring severely ill children, which may detrimentally impact the ongoing post-pandemic care of children with severe lower respiratory infections.

## Introduction

The SARS-CoV-2 virus causes coronavirus disease (COVID-19) and has led to over 6.9 million deaths worldwide [1]. The disease burden of COVID-19 in Sub-Saharan Africa, especially among pediatric patients, is not fully reported. While data from high-income countries report pediatric mortality rates among hospitalized children at <1%, the reported rates in sub-Saharan Africa are as high as 8.3% [2-4]. This represents a potential excess mortality of 23 out of 100 persons higher than the global average [4].

Lesotho is a landlocked country in southern Africa with 2.2 million people and a life expectancy of 55 years [5]. It suffers from the second-highest incidence of tuberculosis and second-highest prevalence of human immunodeficiency virus (HIV) globally [6,7]. Lesotho’s health system lacks hospital capacity in both intensive care and high-dependency units [8-10]. The health system is based on nurse-led primary healthcare centers with one secondary-level, physician-led hospital in each of the 10 districts. There is one public tertiary care referral hospital with a level 3 intensive care unit and invasive mechanical ventilation capabilities [11].

Lesotho confirmed its first case of COVID-19 on May 13, 2020 [12]. As of May 16, 2023, over 766 million COVID-19 cases, and almost seven million deaths have occurred globally [1]. A COVID-19 readiness assessment of Lesotho in 2020 demonstrated significant gaps in COVID-19 preparedness and oxygen infrastructure, including limited use of pulse oximetry, oxygen capacity, shortages in personal protective equipment, and delays in transport [8]. The COVID-19 pandemic prompted rapid escalation in medical oxygen resources increased usage of pulse oximetry for screening and monitoring, increased oxygen capacity, and the development of two dedicated national COVID-19 treatment centers. Treatment centers had greater human resources, more intensive patient monitoring, and oxygen and advanced respiratory care utilizing high-flow nasal cannula (HFNC) oxygen capabilities.

To date, most COVID-19 treatment efforts and oxygen capacity expansion have been focused on adults with limited pediatric focus. To address these deficits, the United States Agency for International Development (USAID) funded Jhpiego Lesotho, an affiliate of Johns Hopkins University, in collaboration with the Lesotho Ministry of Health, to expand current COVID-19 support to children. They did this through pediatric-focused COVID-19 healthcare worker training and the development of contextually appropriate algorithms for pediatric COVID-19 referral, low-flow oxygen, and HFNC oxygen treatment. The overall objective of this study is to better understand the care of pediatric patients at the two national COVID-19 treatment centers during the pandemic, and its potential implications on post-pandemic management of children with acute lower respiratory infections (ALRIs), such as pneumonia and bronchiolitis, in the context of Lesotho. We sought to accomplish this by (1) describing the characteristics and outcomes of pediatric patients referred to COVID-19 treatment centers over the first two years of the pandemic in Lesotho, and (2) evaluating healthcare worker performance in Lesotho on pediatric-specific COVID-19 training.

## Materials and methods

Pediatric COVID-19 cohort

This is a retrospective cohort study of pediatric patients 15 years of age and below hospitalized at the two COVID-19 treatment centers in Lesotho and with known outcomes between May 1, 2020 and April 30, 2022. Fifteen years of age was chosen as the cutoff as this is the age limit for pediatric hospitalization in Lesotho. Children were eligible for hospitalization at the treatment center if they were known COVID-19 positive or suspected of having COVID-19 disease, high risk of severe disease including age <1 year, HIV positive or exposed, having a co-morbidity, or hypoxemic despite four L nasal cannula oxygen. All hospitalized children were included. Demographic and clinical data, including hospital outcomes, were prospectively documented on standardized medical charts by trained healthcare workers. Confirmed cases were SARS-CoV-2 polymerase chain reaction (PCR) or antigen-positive. Hypoxemia was a peripheral oxyhemoglobin saturation (SpO_2_) <94% measured by a pulse oximeter. Severe disease was defined by significant vital sign abnormalities (SpO_2_ <94%, severe tachypnea, severe tachycardia, systolic hypotension, or altered mental status), difficulty breathing, or end-organ dysfunction.

Study Setting and Period

Patients were hospitalized at two hospitals designated as COVID-19 treatment centers: Berea Hospital, a district hospital in Teyateyaneng district in the northern region, and Mafeteng Hospital, a regional hospital in Mafeteng district in the southern region. During the pandemic, Berea Hospital established a 23-bed open high-dependency unit with HFNC oxygen and continuous monitoring capabilities and a 21-bed COVID-19 isolation unit. The high-dependency unit was considered open as it was operated by general physicians. Initially, both wards were supplied oxygen via mobile cylinders and concentrators; by November 2021, piped oxygen was provided by a dedicated PSA oxygen plant. Although staffing levels fluctuated during the study period, those in general levels were at 10 or more doctors and 30 or more nurses overall. Mafeteng Hospital also established a 20-bed COVID-19 isolation unit with oxygen and HFNC oxygen capacity. Oxygen was supplied by mobile cylinders and oxygen concentrators. The isolation unit was staffed by two or more physicians and eight or more nurses in total. Both treatment centers admitted both adult and pediatric patients. Typically, doctors with general physician training reviewed patients one to two times daily, depending on the illness acuity, and directed the overall management of patients. Registered nurses conducted vital signs every four to eight hours, administered medicines and intravenous fluids, and managed low-flow conventional oxygen from oxygen concentrators and cylinders and HFNC (from November 2020) using Airvo™2 (Fisher & Paykel Healthcare, Auckland, New Zealand) devices. One physician was on call at all times but usually offsite during most evening and weekend hours. Nurses were always available in person, with nurse-to-patient ratios ranging from 1:4 to 1:10 at Berea, depending upon the patient’s illness acuity, and from 1:10 to 1:20 at Mafeteng.

Clinical patient-level data were documented during routine programmatic care using standard case reporting forms for the first two years of the COVID-19 pandemic. Lesotho experienced four waves of SAR-CoV-2 infections during this period, and the wave periods were defined as follows: Wave 1 was from March 2020 to November 2020, Wave 2 was from November 2020 to May 2021, Wave 3 was from June 2021 to October 2021, and Wave 4 was from November 2021 to May 2022.

Pediatric COVID-19 educational training program

One-Day Training

Three one-day training sessions were facilitated in person by three physicians (EDM, MR, LG). Training took place at three hospitals: Berea Hospital, Mafeteng Hospital, and Queen Mamohato Memorial Hospital, which is a tertiary referral hospital. All 93 available healthcare workers were invited to participate in any of the training sessions held between March 31 and April 1, 2022, at offsite conference venues near the three hospitals. Training covered epidemiology, pathophysiology, clinical manifestations, case presentations, and treatment of children with COVID-19 infection. The training was intended to build upon prior training that focused mainly on COVID-19 in the adult population.

Pre- and Post-training Examinations of Training Sessions

We administered a 15-question, multiple-choice, paper-based examination immediately before and after the one-day training to evaluate participant baseline and acquired knowledge as an assessment of training effectiveness (Supplemental Material). Participants were asked to complete the examinations in 30 minutes, individually, without the use of training resources. When possible, questions were designed to assess learners’ ability to apply knowledge, not simply convey rote memorization.

Statistical analysis

For patient care data, data clerks electronically entered data from the standardized medical charts from the treatment centers into an encrypted server. We described normally distributed continuous variables with means and standard deviation (SD), non-normally distributed continuous variables by medians and interquartile ranges (IQR), and bivariate or categorical variables with proportions. We used the χ^2^ and Fischer's exact test to assess for any differences in distributions among categorical variables. To evaluate for associations between each variable and death, we used the independent sample t-test and the Mann-Whitney U test for parametric and nonparametric continuous variables and χ^2^ or Fisher's exact tests for categorical variables.

For the educational training program data, we assessed participant performance on both the pre- and post-training examinations. A passing score was considered >80% or 11/14 correct answers. When scoring the examination, one of the 15 questions was eliminated before data analysis due to the lack of question clarity. The outcome of interest was the average change in participant test score between the pre- and post-training examinations. In addition, we sought to analyze if results differed by healthcare worker cadre. We used paired t-tests to evaluate the difference in average scores of participants between the pre- and post-training examinations.

## Results

Pediatric COVID-19 cohort

Demographics

A total of 1,448 patients with confirmed or suspected COVID-19 and known outcomes were hospitalized at the two treatment centers between May 1, 2020, and April 30, 2022. Pediatric patients aged 15 years and below made up only 1.2% (18/1,448) of all hospitalized patients in the treatment centers. Lesotho experienced four waves of SARS-CoV-2 variants during this period. Seven children were admitted to each of Waves 1 and 4, while only two children in each of Waves 2 and 3 (Table 1). Patients had a median age of seven years (IQR: 2-12) and 44.4% (8/18) were female. Twelve of the 18 patients (66.7%) were confirmed as SARS-CoV-2 positive; 22.2% (4/18) were HIV-infected, and 55.6% (10/18) had an unknown HIV status. No patients were vaccinated against COVID-19.

**Table 1:** Characteristics and outcomes of children hospitalized at COVID-19 treatment centers in Lesotho from 2020 to 2022 P-values were calculated using χ2 and Fischer's exact test for categorical variables. The independent sample t-test and the Mann-Whitney U test were used to compare the variables and death for parametric and nonparametric continuous variables. p<0.05 was considered statistically significant. IQR, interquartile range; SD, standard deviation; SpO_2_, arterial oxyhemoglobin saturation. ^1^Defined as 15 years of age or less. ^2^Wave 1, March 2020 to November 2020; Wave 2, November 2020 to May 2021; Wave 3, June 2021 to October 2021; Wave 4, November 2021 to May 2022. ^3^Negative status, SARS-CoV-2 PCR-negative; positive status, SARS-CoV-2 antigen or PCR-positive; suspect status, SARS-CoV-2 antigen-negative with unknown PCR status. ^4^Five missing overall, four alive, and one dead. ^5^Seven missing overall, six alive, and one dead. ^6^15 missing overall, 11 alive, and four dead. ^7^12 missing overall, 10 alive, and two dead. ^8^Defined as any of the following: lower chest wall indrawing, nasal flaring, grunting, or tracheal tugging. ^9^Defined as any of the following: vital sign abnormalities, difficult breathing reported or respiratory distress observed, or end-organ dysfunction or failure. ^10^Eight missing overall, seven alive, and one dead.

Characteristic		Total, N=18	Alive, n=14	Dead, n=4	P value
Age in years	Median (IQR)	7.4 (1.7, 12.2)	8.9 (1.9, 12.8)	4.4 (0.4, 10.0)	0.381
Sex, n (%)	Female	8 (44.4%)	6 (42.8%)	2 (50.0%)	1.000
	Male	10 (55.5%)	8 (57.1%)	2 (50.0%)	
Facility, n (%)	Berea	15 (83.3%)	13 (92.8%)	2 (50.0%)	0.108
	Mafeteng	3 (16.6%)	1 (7.1%)	2 (50.0%)	
COVID-19 Wave Period, n (%)^2^	1	7 (38.8%)	7 (50.0%)	0 (0%)	0.247
	2	2 (11.1%)	1 (7.1%)	1 (25.0%)	
	3	2 (11.1%)	1 (7.1%)	1 (25.0%)	
	4	7 (38.8%)	5 (35.7%)	2 (50.0%)	
COVID-19 status, n (%)^3^	Negative	2 (11.1%)	2 (4.2%)	0 (0%)	1.000
	Positive	12 (66.6%)	9 (64.2%)	3 (75.0%)	
	Suspect	4 (22.2%)	3 (21.4%)	1 (25.0%)	
HIV status, n (%)	HIV uninfected	4 (22.2%)	3 (21.4%)	1 (25.0%)	0.275
	HIV infected	4 (22.2%)	2 (14.2%)	2 (50.0%)	
	HIV unknown	10 (55.5%)	9 (64.2%)	1 (25.0%)	
Vital signs on admission	Temperature (°C), mean (SD)^4^	36.7 (0.4)	36.6 (0.4)	36.9 (0.4)	0.311
	Pulse rate, beats/minute, median (IQR)^5^	124 (105, 133)	126 (96, 131)	118 (105, 148)	1.000
	Respiratory rate, breaths/minute, mean (SD)^6^	35 (11.7)	35 (11.7)	–	–
	Glasgow Coma Scale, mean (SD)^7^	14.3 (1.6)	15.0 (0)	13.0 (2.8)	0.177
Blood sugar on admission, n (%)	Normal	3 (16.6%)	3 (21.4%)	0	1.000
	Low	3 (16.6%)	2 (14.2%)	1 (25.0%)	
	Missing	12 (66.6%)	9 (64.2%)	3 (75.0%)	
Tachycardia for age at admission, n (%)	No	5 (27.7%)	3 (21.4%)	2 (50.0%	0.319
	Yes	10 (66.6%)	9 (75.0%)	1 (33.3%)	
	Missing	3 (16.6%)	2 (14.2%)	1 (25.0%)	
Fast breathing for age at admission, n (%)	No	1 (5.5%)	1 (7.1%)	0	1.000
	Yes	2 (11.1%)	0	2 (14.2%)	
	Missing	15 (83.3%)	11 (78.5%)	4 (100.0%)	
Respiratory distress at admission, n (%)^8^	No	17 (94.4%)	14 (100.0%)	3 (75.0%)	0.222
	Yes	1 (5.5%)	0	1 (25.0%)	
Severe or critical condition at admission, n (%)^9^	No	12 (66.6%)	11 (78.5%)	1 (25.0%)	0.083
	Yes	6 (33.3%)	3 (21.4%)	3 (75.0%)	
SpO_2_ on admission^10^	Median (IQR)	94% (88%, 97%)	95% (88%, 98%)	91% (82%, 96%)	0.516
SpO_2 _<24 hours of admission, n (%)	>94%	6 (33.3%)	5 (35.7%)	1 (25.0%)	0.500
	<94%	4 (22.2%)	2 (14.2%)	2 (50.0%)	
	Missing	8 (44.4%)	7 (50.0%)	1 (25.0%)	
SpO_2 _during hospitalization, n (%)	>94%	10 (55.5%)	10 (71.4%)	0	0.023
	<94%	8 (44.4%)	4 (28.5%)	4 (100.0%)	
Respiratory support during hospitalization, n (%)	None	9 (50.0%)	9 (64.2%)	0	0.007
	Low flow oxygen	7 (38.8%)	5 (35.7%)	2 (50.0%)	
	High flow nasal cannula oxygen	2 (11.1%)	0	2 (50.0%)	
Hospital length of stay in days	Median (IQR)	5 (3, 10)	5 (3, 10)	5 (1, 10)	0.747

Oxygen Utilization

The median SpO_2_ on presentation was 94% (n=10, IQR: 88-97%) (Table 1). Four of 18 (22.2%) children were hypoxemic (SpO_2_ <94%) in the first 24 hours of admission and eight (8/18, 44.4%) were hypoxemic at any point during their hospitalization (Table 1). Despite hypoxemia, only one child was recorded as having shortness of breath, and no other signs of respiratory distress, including head nodding, nasal flaring, grunting, tracheal tugging, intercostal recessions, or lower chest indrawing on presentation, were noted. Half (9/18) of the patients received oxygen therapy, which increased in frequency across waves. Specifically, none of the seven children admitted during Wave 1 were treated with oxygen compared to 100% (4/4) of children in Waves 2 and 3 and 71.4% (5/7) in Wave 4 (p=0.006). By Wave 4, both treatment centers had HFNC capabilities and algorithms for pediatric HFNC administration. Only two children, ages 10 months and 12 years, were treated with HFNC oxygen during the pandemic period. Both children were HIV-infected, COVID-19 positive with severe illness on presentation, and died.

Mortality

Four of the 18 (22.2%) patients died. Although total cases were small, there were no differences between those who survived and those who died in terms of age, sex, COVID-19 status, facility, or wave period (Table 1). Among patients who survived, only 28.6% (4/14) were hypoxemic during hospitalization compared to 100% (4/4) of children who died (p=0.023). All 10 children without hypoxemia survived. Severe illness on presentation was higher among children who died (3/4, 75%) than in those who survived (3/14, 21.4%) (p=0.083).

Pediatric COVID-19 educational training program

A total of 82 attendees completed the pre-training examination and 79 completed the immediate post-training examination. Seventy-six participants completed both examinations. Nurses comprised 56/82 (68.3%) of the participants, physicians 14/82 (17.1%), and other health care worker cadres 12/82 (14.6%). Other HCWs included nursing assistants, biomedical technicians, laboratory technicians, radiographers, pharmacists, and a unit manager, none of whom provided unsupervised direct patient care.

Pre-training Examination Performance

Among all participants, the mean number of correct answers on the 14-question pre-training examination was 6.2 or a mean of 45.1% (95% CI, 41.0-48.2) correct. The lowest score of 3/14 was achieved by four participants while a passing score of >80% was generated by only two participants. The average percentage score on the pre-training examination was higher among the 14 physicians (53.5%) than the 60 nurses (43.1%) (p=0.0260).

Post-training Examination Performance

**Figure 1:**
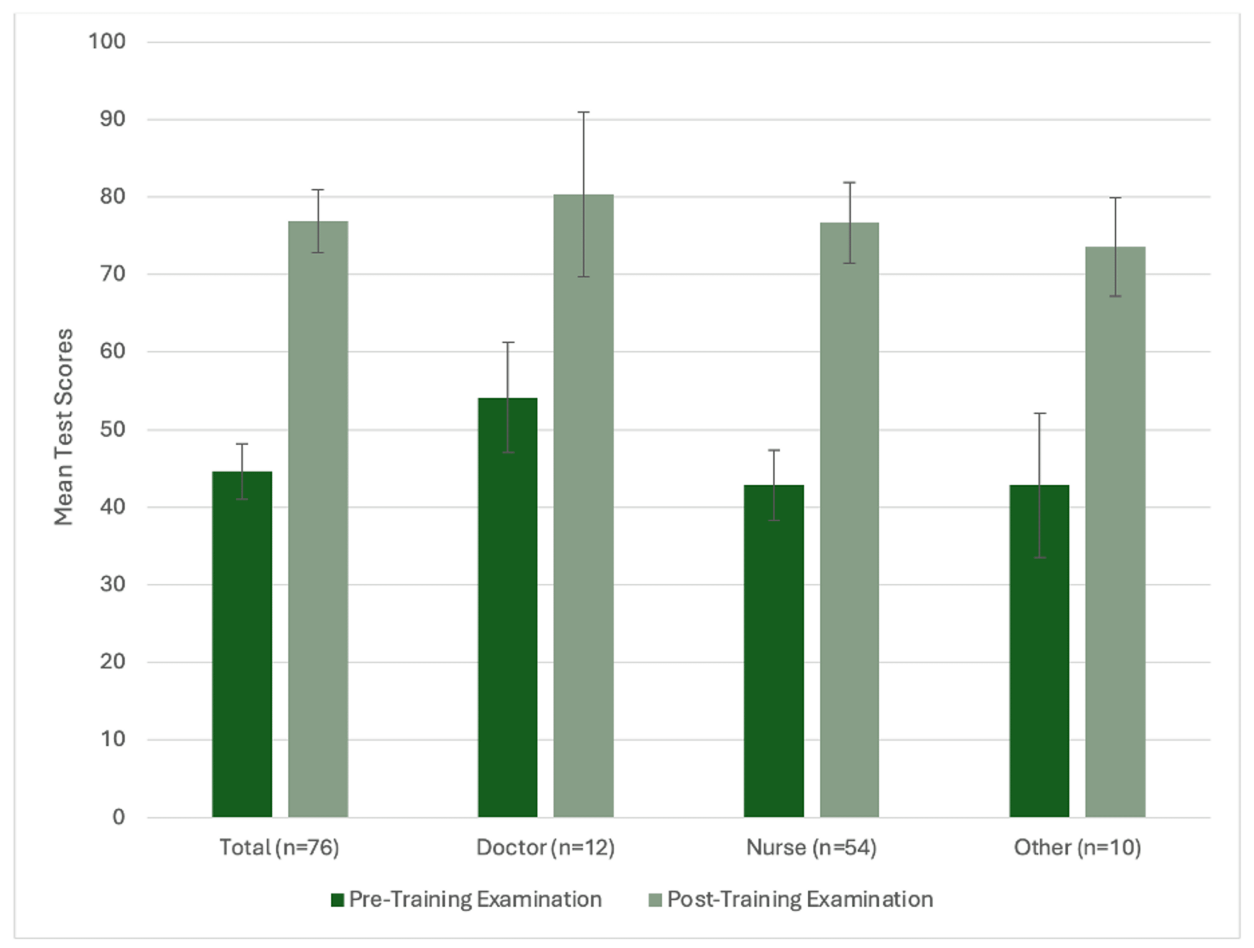
Pediatric COVID-19 pre-training and post-training examination scores by cadre Bars represent 95% confidence intervals. Doctors and nurses comprised 66/76 participants. The remaining 10 participants included other hospital staff such as radiology and lab technicians.

The mean percentage score on the post-training examination among 76 participants was 76.9% (95% CI: 72.8, 80.9) correct. Although the overall percentage of participants that achieved a passing score >80% on the post-training examination was 50.0% (38/76), there was a 32.2% (95% CI: 27.7%, 36.6%; p<0.001) increase in the average post-training examination score, compared to the pre-training score, among the 76 participants that completed both examinations. A perfect score of 100% was achieved by 11.8% (9/76) of participants, while 7.8% (6/76) of participants scored <50%. There was no difference in the average post-training examination scores between healthcare worker cadres (p=0.231) (Figure 1). Importantly, there was also no difference in the average change in scores between the pre- and post-training examinations when stratified by the 12 physicians (mean change: 26.1%, 95% CI: 15.5, 36.8; p=0.0002) and 54 nurses (mean change: 33.8%, 95% CI: 28.1, 39.5; p<0.0001) (p=0.2371) that completed both examinations.

General Oxygen Questions

While most of the training and questions were focused on COVID-19-specific knowledge, three questions assessed general oxygen and pulse oximetry knowledge. One question asked participants to identify valid pulse oximetry plethysmography waveforms, which are utilized by most handheld and monitor-based pulse oximeters to evaluate SpO_2_ measurement quality in Lesotho. Despite routine use of pulse oximetry monitoring that utilizes waveform assessment in all three facilities, only 63.2% (48/76) of participants answered correctly on the pre-training examination. Performance improved by an average of 15.8% (95% CI: 2.8, 28.7) with 78.9% (60/76) correctly answering this question on the post-training examination (p=0.0176). Similarly, for one question about identifying fast breathing for age in children, just 52.6% (40/76) answered correctly on the pre-training examination and 67.1% (51/76) on the post-training examination, reflecting a mean of 14.4% (95% CI: 0.6, 28.3; p=0.0403) improvement between examinations among participants that completed both tests.

## Discussion

The COVID-19 pandemic prompted a rapid push to increase pulse oximeter availability and oxygen capacity worldwide, including in Lesotho. With funding from the USAID RISE program and support from Jhpiego, the Lesotho Ministry of Health, and other private partnerships, Lesotho was able to significantly increase the capacity to treat ALRIs through advances in pulse oximeter access, oxygen infrastructure, equipment, policies, and human resource capacitation. Pandemic efforts, while targeted at COVID-19, have broader applicability in the treatment of all causes of hypoxemia, primarily other ALRIs, as countries transition into a post-pandemic phase. For example, pneumonia remains the leading infectious cause of under-5-year-old mortality worldwide [13], emphasizing the need for improved care of ALRI, including expanded oxygen capacity, even in non-pandemic times. Experience in caring for patients with COVID-19 can also provide insight into health system preparedness for future respiratory pandemics.

In this report, we describe the experience with pediatric COVID-19 in Lesotho, including a cohort of pediatric patients hospitalized in two national COVID-19 treatment centers and training designed to capacitate healthcare workers in caring for pediatric patients with COVID-19. With the establishment of the COVID-19 treatment centers, patients of all ages with suspected or confirmed COVID-19 with severe or critical disease or at high risk of decompensation due to their comorbidity profile were recommended for referral to either treatment center. A positive SARS-CoV-2 PCR or antigen test was not required for referral; therefore, these criteria should have resulted in most children with severe ALRI receiving care at the treatment centers where oxygen resources and augmented staffing levels were concentrated. Centralization of critical care resources is common in low- and middle-income countries and intensive care units (ICU) and high-dependency units are often only at tertiary hospitals in urban areas [14]. Therefore, in keeping with WHO recommendations, many countries implemented triage and referral algorithms during the pandemic to identify patients at the highest risk of severe disease and to promote timely referral to centers with greater resources [15]. Even when triage systems and referrals are in place, transportation and resource availability at referral centers are often limited. The WHO reports that the African region has fewer than 5,000 ICU beds and globally 90% of all low-income countries have fewer than 10 medical doctors per 10,000 population [15]. Despite broad referral recommendations in Lesotho, we reported that only 18 pediatric patients were hospitalized over a 24-month period, making up just 1.2% of all patients, suggesting notable challenges in executing triage and referral recommendations.

Data from primarily high-income countries reveal between 1% and 5% of all COVID-19 cases occur in children with <5% of children having severe disease [3,16,17]. While one study demonstrated children with comorbidities have a relative risk of mortality of 2.8, overall COVID-19 mortality is still very low at <1% of hospitalized children [3,4,16]. In a sub-Saharan African cohort, mortality was significantly higher at 8.3% of hospitalized children with COVID-19 [4]. This is in part due to higher rates of comorbidities, with 25% of hospitalized children having >1 comorbidity including hypertension, chronic lung disease, and hematologic disorders. Amongst our cohort, pediatric patients made up a small percentage of overall patients. However, our cohort also had significantly higher mortality (22%) than reported the <1% globally or ~8% in other Sub-Saharan African contexts. As previously noted, such small numbers and high mortality amongst our cohort may reflect failures in the referral process including late referral, only the sickest children being referred, difficulty in recognizing illness severity, or inadequate physical or human resources to treat pediatric patients.

We observed that none of the 18 pediatric patients hospitalized were recorded as having signs of increased work of breathing such as head nodding, chest indrawing, tracheal tugging, or grunting. Overall, a systematic review of the clinical signs of COVID-19 in children found that among all patients - both inpatient and outpatient - only 3.5% experienced signs of respiratory distress [18]. However, when present, signs of respiratory distress predict severe disease with up to 87% of pediatric patients with severe disease having clinical features of dyspnea [19]. Given our study population of severely ill hospitalized children with high mortality, it is surprising that no child in this study was reported to have signs of respiratory distress. This could be due to incomplete documentation. However, it also suggests that signs of illness severity in children may have been missed by healthcare workers. This hypothesis is supported by the low test scores on the pediatric COVID-19 knowledge assessments. There is a strong association between hypoxemia and signs of respiratory distress, and therefore it is critical for providers to be able to identify respiratory distress and closely monitor SpO_2_ levels [20].

In addition to the creation and outfitting of dedicated treatment centers, healthcare worker trainings were held to capacitate doctors, nurses, and other healthcare staff on pediatric COVID-19. These were part of a series of respiratory trainings and simulations throughout the pandemic including both adult and pediatric cases. Given previous sessions, the pediatric COVID-19 training presumed a fundamental knowledge base in caring for children with respiratory illnesses. Training content primarily focused on COVID-19-specific issues. We found that, similar to the test scores for adult COVID-19 training in Lesotho [21], scores on both the pre- and post-training examinations for pediatric COVID-19 training were low. When looked at individually, test scores on generalized respiratory questions not specific to COVID-19 were also modest. Scores on the post-training examination increased across all healthcare worker cadres, demonstrating short-term knowledge acquisition. However, overall modest scores on the post-training test may suggest a mismatch between content and/or approach with trainee background and experience levels in acute respiratory care. The providers’ pre-training knowledge base may have been insufficient to fully engage with the training.

When taken together, the pediatric cohort and healthcare worker knowledge assessments suggest limited experience with and expertise in caring for pediatric patients with ALRI. However, progress was noted in both the number of children treated with oxygen across waves and post-training examination scores. The pediatric cohort’s use of pulse oximetry, low-flow oxygen, and HFNC oxygen aligns with the growing national oxygen capacity over the two-year period. None of the children in the first wave of COVID-19 were treated with oxygen and HFNC was not implemented and utilized for pediatric patients until Wave 4. Vital sign measurements, including consistent recording of pulse oximetry, also increased in subsequent waves of COVID-19, likely due to both increased availability of pulse oximeters and an increase in healthcare worker familiarity with and reliance on pulse oximetry in guiding oxygen administration. This momentum initiated by the COVID-19 pandemic is critical to continue to advance pediatric respiratory care to decrease mortality and create long-term benefits.

The retrospective nature of our cohort and rapid deployment of training to meet pandemic needs comes with several limitations. First, data were only available from patients who were hospitalized at the treatment centers, which does not fully reflect pediatric COVID-19 care nationwide. Second, our clinical data were collected during routine care, and several variables early in the study period had high missingness, although this improved over the study period and in part reflected a strengthening of the overall quality of care at the treatment centers. For example, most patients who were admitted during wave one did not have a full set of vital signs recorded compared to no missing vital sign data in wave four. Third, to deploy training quickly, we made assumptions about the baseline educational background knowledge of HCWs, and trainings were designed to teach providers from a wide range of training backgrounds. To refine future pediatric respiratory training, a deeper understanding of current knowledge backgrounds and gaps is needed.

## Conclusions

While the COVID-19 pandemic led to a nationwide ramp-up of oxygen capacity, the limited pediatric referral and healthcare worker knowledge we observed in this study highlight continued deficits in recognition and care for pediatric respiratory illnesses in Lesotho. National COVID-19 treatment center data indicates a low burden of severe pediatric COVID-19 disease in Lesotho. However, HCW knowledge gaps suggest deficiencies in identifying and referring severely ill children, which may be important and could detrimentally impact the ongoing post-pandemic care of children with severe lower respiratory infections. We recommend policy, donor, and post-pandemic emphasis on increasing and strengthening pediatric hypoxemia identification and oxygen capacity both to reduce global mortality from ALRI and to prepare for future respiratory pandemics that differentially affect children.

## Figures and Tables

**Figure 2:**
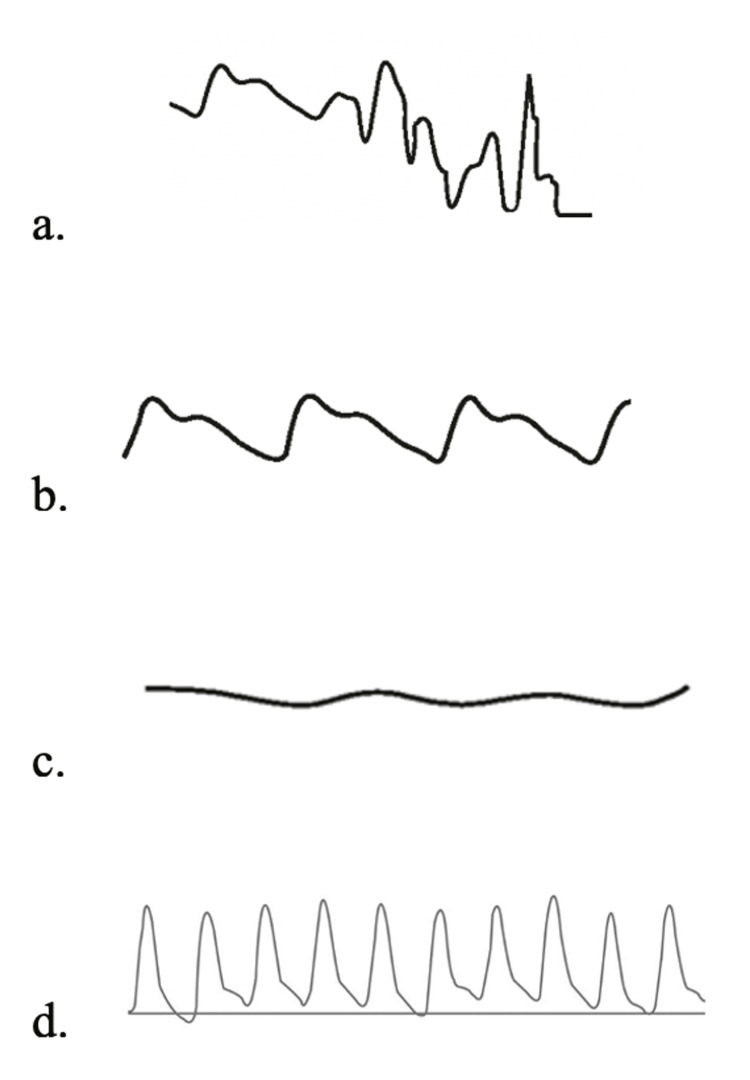
Examples of Waveforms

## Appendices

1.     What is the most common presenting symptom of acute COVID-19 infection in young children? (choose one answer)

a.     Sore throat

b.    Loss of taste

c.     Fever

d.     Diarrhea

e.     Shortness of Breath

2.     What is the best diagnostic test for acute COVID-19 infection in children? (choose one answer)

a.     No testing is needed, it is a clinical diagnosis

b.     Chest X-Ray

c.     Blood testing: White Blood Cell Count

d.     Blood testing: Erythrocyte Sedimentation Rate

e.     COVID-19 PCR Test

3.     True or False: It is possible to test negative despite being infected with the COVID-19 virus. (choose one answer)

a.     True

b.     False

4.     True or False: It is important to give every child suspected of having COVID-19 antibiotics regardless of disease severity. (choose one answer)

a.     True

b.     False

5.     The most important treatment for MIS-C (Multisystem Inflammatory Syndrome in Children) is: (choose one answer)

a.     Diuretics

b.     Steroids

c.     Remdesivir

d.     Antiretrovirals

e.     Antibiotics

6.     Which age group had the highest mortality among children and adolescents hospitalized for COVID-19 in sub-Saharan Africa? (choose one answer)

a.     0-1-year-olds

b.     1-4-year-olds

c.     5-9-year-olds

d.     10-14-year-olds

e.     15-19-year-olds

7.     True or False - Most children hospitalized for COVID-19 in sub-Saharan Africa had comorbidities (i.e. asthma, HIV, SAM, obesity). (choose one answer)

a.     True

b.     False

8.     Which are the two most common ways COVID-19 is transmitted? (select two answers)

a.     Fecal-oral

b.     Droplet

c.     Contact

d.     Airborne

9.     A previously healthy 10-year-old boy presents to the hospital with 4 days of fever, cough, and shortness of breath. A COVID-19 antigen is collected on admission and positive. After two days the child is now doing well and discharged home. The mom asks how long the child should be isolated from the uninfected household members. Per WHO guidelines you tell her (choose one answer):

a.     He has already completed the 5 days of recommended isolation.

b.     10 days from when his symptoms developed

c.     10 days from when he tested positive

d.     14 days from his likely exposure

10.  Which of the following is true regarding multisystem inflammatory syndrome in children (MIS-C) (circle all answers that apply).

a.     Approximately 10% of children infected with SARS-CoV-2 will go on to develop MIS-C

b.     It most commonly affects children with multiple existing comorbidities

c.     It is caused by a dysregulated immune system attacking multiple organ systems

d.     It is most often seen in children younger than 2 years of age

11.  What is the oxygen saturation threshold below which healthcare providers should start low-flow oxygen for a paediatric COVID-19 suspect or confirmed cases? (choose one answer)

a.     <98%

b.     <94%

c.     <90%

d.     <85%

12.  Below are examples of pulse oximeter waveforms (Figure 2). Please select which waveform(s) indicate a quality oxygen saturation measurement (choose one answer).
 

e.     a & d

f.      b & d

g.     a & c

13.  If dexamethasone is used for the treatment of a child with COVID-19, what is the recommended dose? (choose one answer)

a.     6mg (regardless of weight)

b.     0.15mg/kg/dose

c.     6mg/kg/dose

d.     0.6mg/kg/dose

14.  You have counted an 11-month-old child’s respiratory rate to be 52 breaths/minute. This respiratory rate is normal? True or false. (choose one answer)

a.     True

b.     False

15.  What flow rate of high-flow oxygen is recommended for a child weighing 10kg? (choose one answer)

a.     0.5 L/kg/min

b.     3 L/kg/min

c.     5 L/kg/min

d.     2 L/kg/min
